# Bitter or not? BitterPredict, a tool for predicting taste from chemical structure

**DOI:** 10.1038/s41598-017-12359-7

**Published:** 2017-09-21

**Authors:** Ayana Dagan-Wiener, Ido Nissim, Natalie Ben Abu, Gigliola Borgonovo, Angela Bassoli, Masha Y. Niv

**Affiliations:** 1 Institute of Biochemistry, Food Science and Nutrition, The Robert H. Smith Faculty of Agriculture, Food, and Environment, The Hebrew University of Jerusalem, Rehovot, 76100 Israel; 2 The Fritz Haber Center for Molecular Dynamics, The Hebrew University of Jerusalem, Jerusalem, 91904 Israel; 3 DeFENS-Department for Food, Environmental and Nutritional Sciences, University of Milan, Via Celoria 2, Milano, 20133 Italy

## Abstract

Bitter taste is an innately aversive taste modality that is considered to protect animals from consuming toxic compounds. Yet, bitterness is not always noxious and some bitter compounds have beneficial effects on health. Hundreds of bitter compounds were reported (and are accessible via the BitterDB http://bitterdb.agri.huji.ac.il/dbbitter.php), but numerous additional bitter molecules are still unknown. The dramatic chemical diversity of bitterants makes bitterness prediction a difficult task. Here we present a machine learning classifier, BitterPredict, which predicts whether a compound is bitter or not, based on its chemical structure. BitterDB was used as the positive set, and non-bitter molecules were gathered from literature to create the negative set. Adaptive Boosting (AdaBoost), based on decision trees machine-learning algorithm was applied to molecules that were represented using physicochemical and ADME/Tox descriptors. BitterPredict correctly classifies over 80% of the compounds in the hold-out test set, and 70–90% of the compounds in three independent external sets and in sensory test validation, providing a quick and reliable tool for classifying large sets of compounds into bitter and non-bitter groups. BitterPredict suggests that about 40% of random molecules, and a large portion (66%) of clinical and experimental drugs, and of natural products (77%) are bitter.

## Introduction

Bitter taste is a basic taste modality, which is believed to have evolved to protect animals from consuming toxic food^1^. It was estimated that the number of bitter compounds is in the thousands or tens of thousands^2^. We have established BitterDB http://bitterdb.agri.huji.ac.il/dbbitter.php, a database which now holds about 700 compounds that were reported to have bitter taste or to activate at least one human bitter receptor in cell-based assays^3^. BitterDB includes structurally diverse compounds such as ions, peptides, alkaloids, polyphenols, glucosinolates and more. The full repertoire of molecules that activate bitter taste receptors is currently unknown. Furthermore, the size of the bitter chemical space, the abundance of bitter compounds in chemical space (both natural and synthetic) are currently unknown.

The bitter taste is recognized in human by 25 G-protein-coupled receptors (called T2Rs or TAS2Rs). These receptors are expressed in the oral cavity, as well as in the gastrointestinal tract, the upper airways, the heart and in additional tissues^4–6^. Indeed, T2Rs have physiological roles in the digestive process^7^, affecting respiration and activating the immune system^8,9^. T2Rs have been suggested as novel therapeutic targets for asthma^10^ and respiratory infections^8,11^.

Prediction of bitterness has therefore several practical implications: predicting bitter compounds within the human metabolome may suggest yet unknown endogenous ligands of T2Rs. Identifying bitterness of approved drugs may be useful for their repurposing for novel indications^12^. On the other hand, bitterness of drug molecules presents compliance problems^13,14^ and early flagging of potential bitterness of a drug candidate may help its further development. Bitterness prediction is also important for the food industry, for example indicating if key ingredients are likely to be bitter and therefore require application of masking procedures^15,16^.

The main challenge in computational bitterness prediction arises from the chemical diversity of bitter compounds, while only minor differences between bitter and non-bitter compounds exist in some cases^17^. Therefore, most of the predictive models focused on specific families of bitter compounds^18^ such as cynaropicrin and grosheimin derivatives^19,20^, and benzenesulfamates^21^. Bitterness of peptides was also examined in several past studies^22,23^.

Several successful ligand-based and structure-based approaches predict bitter molecules that activate a specific bitter receptor and were successful in predicting ligands of bitter receptors with a large number of known ligands^24–26^.

Methods for predicting bitterness using machine learning approaches were developed to address bitterness prediction not limited to a particular T2R or chemical family: Rodgers and colleagues used a dataset of 649 bitter and 13,530 randomly selected molecules which were approximated as non-bitters, to develop a Naïve Bayes classifier based on circular fingerprints (MOLPRINT2D^27^) and information-gain feature selection^16^. The proprietary classifier and dataset are unavailable to the research community.

Huang *et al*.^28^ used support vector machine (SVM) algorithm to build BitterX tool, which is based on physicochemical descriptors of the compounds and can be used to predict bitterness. In combination with receptors descriptors it predicts also which of the T2Rs the compound may activate. The positive set was built from BitterDB^3^ and additional manually curated compounds (~500 in total); the negative set was comprised mainly from representative structures of compounds which were not described as ‘bitter’ in the Available Chemicals Directory (ACD, http://www.accelrys.com). BitterX is available via http://mdl.shsmu.edu.cn/BitterX/.

In the current study we developed a general predictor applicable to diverse chemical families, to allow answering the following questions: I) For any given compound, is the compound likely to be bitter or non-bitter? II) In a library of compounds (i.e. food-derived, drug-related, natural, etc.) – what percent of compounds is expected to be bitter? In other words, what is the abundance of bitter compounds in a given chemical space? Finally – III) Are there characteristic properties of bitter vs. non-bitter compounds?

Below we describe the development and applications of the machine learning method BitterPredict that addresses these questions.

## Results

A key step in development of machine learning predictor is the construction of true positive and true negative datasets. Below we describe these datasets and analyze their properties in comparison to the ChEBI^29^ database, which is taken as a set of random molecules.

### Chemical Entities of Biological Interest (ChEBI) set

A dataset of tens of thousands ‘small molecular entities’. ChEBI stands for ‘**Ch**emical **E**ntities of **B**iological **I**nterest’. It is a freely available database of ‘small molecular entities’, developed at the European Bioinformatics Institute (EBI)^29^. The compounds and other molecular entities within this dataset are either products of nature or synthetic products used to intervene in the processes of living organisms. After data curation, 41,132 molecules were ready for prediction with 29,340 molecules in Bitter Domain. This set was used as “random” set, aiming representing a general chemical space.

### The positive (bitter) set

The major resource for the positive set includes 632 molecules from BitterDB^3^ and additional 59 compounds from a recent study by Rojas *at el*.^30^.

**Figure 1:**
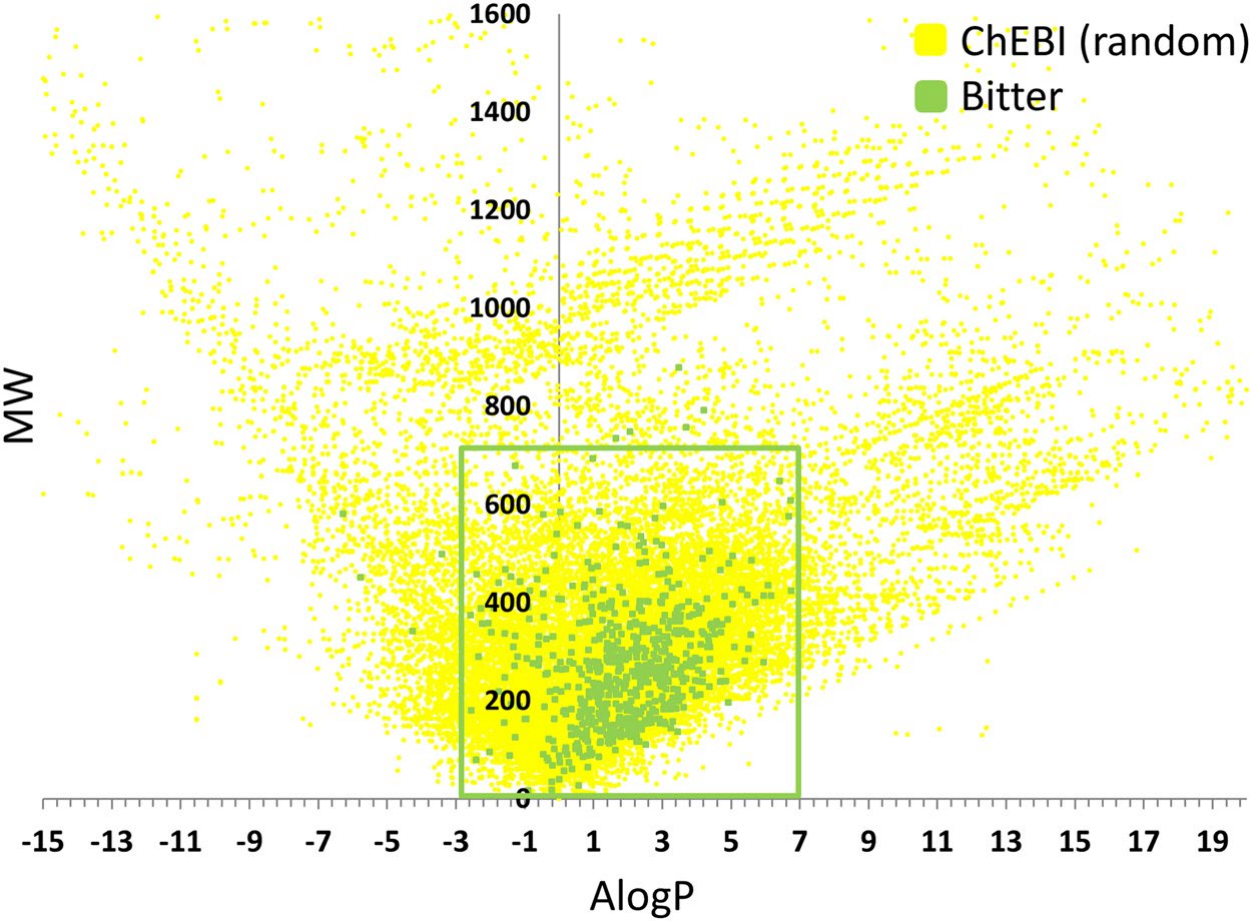
“Bitter Domain”: scatter plot of AlogP vs MW of the random molecules and the positive set molecules. The green rectangle represents the Bitter Domain which is defined by −3 = <AlogP < = 7 and MW < = 700, and includes 97% of the bitter molecules.

691 (97%) of the positive set molecules have molecular weight (MW) < = 700 and hydrophobicity (AlogP) range −3 < = AlogP < = 7 (Fig. 1). Therefore, the predictive model was restricted to this “bounding box”^31^. Molecules outside this domain can be assumed to be non-bitter, unless a more specialized approach (such as ligand-based approach trained on the specific chemical family) suggests otherwise. We named this applicability domain “Bitter Domain”. All datasets used for training and prediction were first filtered to exclude molecules outside this domain.

### The negative set

To the best of our knowledge, no publically available set of non-bitter molecules exists, and such data is not easy to retrieve. Previous studies used random molecules^16^ or undisclosed in-house sets^28^. We established a dataset of ~2,000 non-bitter molecules (all within the Bitter Domain), composed from three subsets: non-bitter flavors, sweet molecules, and tasteless molecules, as detailed below.

Non-bitter flavors subset**:** The non-bitter flavors subset comprises 1,360 ‘likely not bitter’ molecules that were collected from Fenaroli’s Handbook of Flavor Ingredients^32^ in an automated fashion. Compounds were considered as non-bitter if the word bitter did not appear in its description. In addition to flavors, this subset also contains 56 molecules which were cited in TOXNET^33^ as tasteless and 35 additional non-bitter molecules that were manually extracted from the literature^17,34,35^.

Sweet and tasteless subsets were built from sweet (not reported to be bitter) (336) and tasteless (130) compounds recently reported by Rojas *at el*.^30^.

### Random molecules

Random molecules were represented by molecules from Chemical Entities of Biological Interest (ChEBI) dataset^29^ (see Methods). The set includes more than 40,000 molecules, among them almost 30,000 compounds within the Bitter Domain. This set was used as reference set, representing a general and wide chemical space.

### Principal component analysis (PCA)

**Figure 2:**
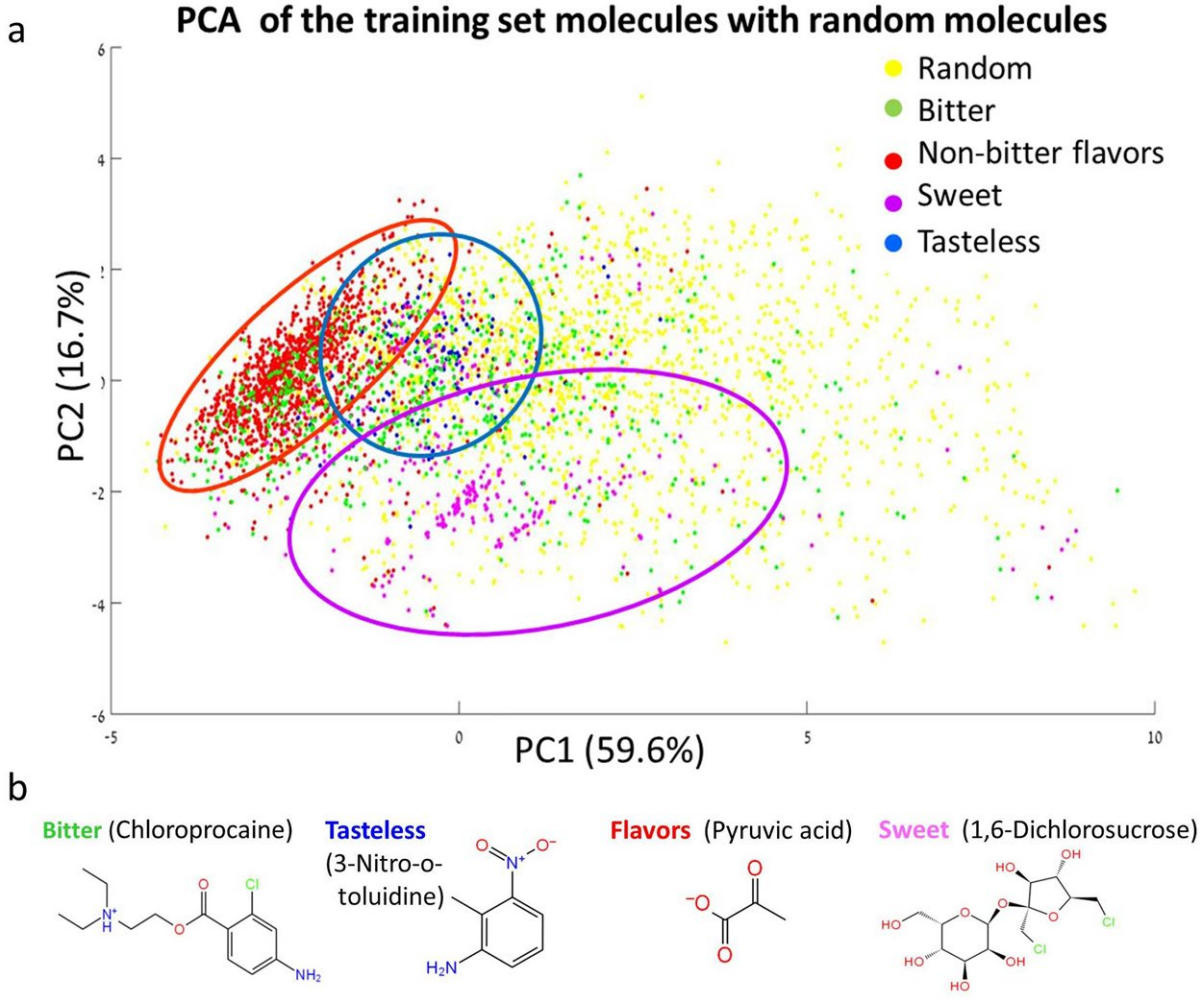
Training data chemical space (**a**) PCA of the negative set, positive set and random molecules within the Bitter Domain (for PCA details see Methods). The bitter molecules (green) spread widely inside the Bitter Domain. Each non-bitter sub set covers distinct sub-space; however the combined non-bitter set covers almost all the domain, though not uniformly distributed. Principle component 1 (PC1) and PC2 explain ~60% and ~17% of the variation, respectively. (**b**) Examples of molecule structures from the positive set and the different negative sub sets.

PCA using 12 basic physicochemical properties of the negative set, positive set and random molecules within the Bitter Domain, shows that the negative set covers almost all of Bitter Domain, and that each negative subset (flavors, sweet, and tasteless) captures a partial region of the chemical space in the Bitter Domain (Fig. 2).

Preliminary testing indicated that the negative set has a dominant effect on the quality of prediction (Supplementary Table S2 shows prediction results using only the non-bitter flavors as the negative set, causing over-prediction of bitterness). The low variance in properties of the non-bitter flavor subset (see Supplementary Fig. S1 for histograms) is likely due to the fact that the majority of molecules in this set are volatile molecules, most of which are relatively small.

### Molecular descriptors

Due to the widely assumed connection between bitterness and toxicity^36^, ADME/Tox (absorption, distribution, metabolism, excretion and toxicity) descriptors from the QikProp package (version 4.6 Schrödinger, LLC, New York, NY, 2015) were used. The QikProp package predicts physically and pharmaceutically significant properties of organic molecules based on the full 3D molecular structure. Ten additional basic physiochemical descriptors which are not part of the QikProp package were also used (see Methods for details).

Bivariate statistical analysis of the training data with selected QikProp descriptors indicated combined properties ranges which are enriched with bitter molecules, such as medium-low skin permeability (QPlogKp) and medium-high hydrophobic component of total solvent accessible surface area (FOSA). The analysis also indicated that bitter molecules are predicted to have low MDCK cell permeability and low predicted brain/blood partition coefficient (QPlogBB) compared to the non-bitter molecules (some of the data is shown in in Supplementary Fig. S2), supporting the idea that QikProp properties are useful for the classifier.

### Algorithms

AdaBoost with decision trees as weak learners^37^ is an adaptive ensemble method, in which the decision trees are built sequentially, by learning from mis-classified samples of the former decision tree. The different sizes of the bitter and non-bitter datasets, as well as different sizes of subsets within the non-bitter, were balanced via the initial observation weight vector (see Methods for details).

### Model performance

In order to avoid overfitting and to get a clear picture of the model performance, the input data was divided randomly to 70% training set and to 30% hold out test set. This split ensures the original proportions in both the training and the test sets. Several parameters were adjusted in respect to the test set performance (for details see Methods).

The classification performance parameters on training and test sets are listed in Table 1. The sensitivity and specificity on the training set were 0.91 and 0.94 respectively and on the test set 0.77 and 0.86 respectively. These results correspond to score threshold of zero (samples with prediction scores larger than zero are classified as bitter and samples with score smaller than zero as non-bitter). The performance on the subsets of the non-bitter molecules is also listed in Table 1, suggesting average specificity >0.8. This analysis shows that the model offers a good classification to bitter and non-bitter molecules.

**Table 1:** Results on training and test sets.

Set	Pos	Neg	TP	FP	TN	FN	Sensitivity TP/TP + FN	Specificity TN/TN + FP	Accuracy TP + TN/Pos + Neg
Training	484	1,343	439	86	1,257	45	0.91	0.94	0.93
Test	207	574	159	83	491	48	0.77	0.86	0.83
Sweet		100		17	83			0.83	
Tasteless		39		7	32			0.82	
Non-bitter flavors		435		59	376			0.86	

Pos- number of positive molecules in the set. Neg- number of negative molecules in the set. TP- number of positive (bitter) molecules correctly classified. FP- number of negative (non-bitter) molecules incorrectly classified. TN- number of negative (non-bitter) molecules correctly classified. FN- number of positive molecules (bitter) incorrectly classified. Sensitivity TP/(TP + FN). Specificity TN/(TN + FP).

**Figure 3:**
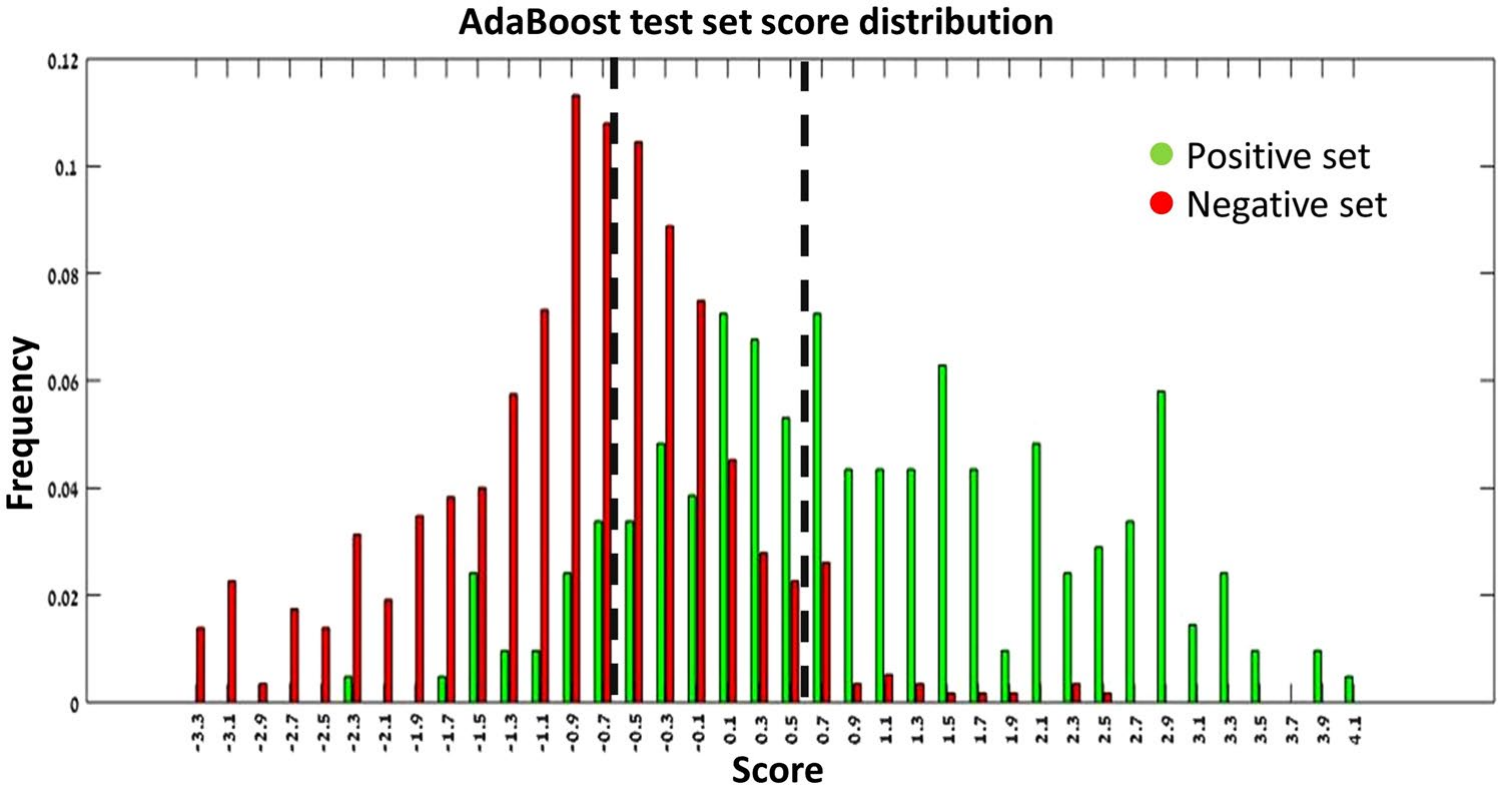
AdaBoost prediction score distribution on the test set: histogram showing the prediction score distribution on the test set. Dashed lines indicate thresholds for more reliable predictions; above score 0.6 and beneath −0.7 false positive and false negative rates respectively, are low.

In some cases the goal may be obtaining high sensitivity, while specificity is less important; in other cases higher specificity is most needed. Changing the threshold score which determines bitter/non-bitter decision can be used to fine-tune the predictor for the needed purpose. Test set compounds with prediction scores greater than 0.6 lead to false positive rate (FP/(FP + TN)) lower than 0.05 and sensitivity above 0.5. Prediction scores less than −0.7 lead to false negative rate (FN/(FN + TP)) lower than 0.01 and specificity above 0.5 (see Fig. 3). Therefore, we suggest a cutoff score > 0.6 when high confidence bitter predictions are required, and a cutoff score of <−0.7 for high confidence non-bitter predictions.

### Validation

Next, BitterPredict performance was evaluated via three approaches:

I) Validation using external sets; II) Validation by literature mining; and III) Validation by taste tests.

I) External sets. In principle, test set overfitting can be caused by selecting the models with the best prediction statistics on the hold out test set^38^. In order to better assess the classifier performance, several external (new) sets were gathered.

#### Bitter New

(molecules collected from the literature and not included in BitterDB or the training set): 29 molecules not included in the training or test sets, were collected from six publications^39–44^. 6 of the molecules lay outside Bitter Domain (MW > 700), 5 of those are tannin compounds (procyanidin B, C and PGG).

The prediction was performed on the remaining 23 structurally diverse compounds (MW ranges from 60 to 600 (g/mol) and AlogP ranges from −1.5 to 6).

BitterPredict correctly predicted 17 out of the 23 molecules (0.74 Sensitivity). 14 of the 17 true positive molecules got prediction scores higher than 0.6. 3 of the 6 bitter molecules which were misclassified as non-bitter are ethyl esters, and only one of the six misclassified molecules has a high negative score (<−0.7).

#### UNIMI set

Previously unpublished data from Bassoli lab at the University of Milan (UNIMI). The set contains 64 molecules, including 23 bitter, 33 non-bitter, 4 “unpleasant” and 4 with undefined taste. The “unpleasant” molecules were excluded due to the difficulty to discriminate between bitter and other unpleasant tastes.

This set is very challenging, because it contains molecules which share the same scaffold but elicit different tastes. BitterPredict was correct on 78% of the bitter and 85% of the non-bitter compounds in this set. In the misclassified samples there are 2 molecules for which stereoisomers are present in this set, but with different taste (see Supplementary Fig. S3), exemplifying a case which might be better addressed by specialized predictors.

#### Molecules from the Phytochemical Dictionary

54 bitter and 39 non bitter (mostly sweet) molecules were extracted from the Phytochemical Dictionary book^45^ which includes information about taste of bioactive compounds from plants. These molecules were not part of the training or test sets. 6 of the 54 bitter molecules and 13 of the 39 non-bitter molecules were outside the Bitter Domain. BitterPredict correctly classified 98% of the bitter molecules and 69% of the non-bitter molecules. 14 of the 18 molecules correctly classified as non-bitter, scored <−0.7; 44 of the 48 molecules correctly classified as bitter, scored >0.6.

Table 2 shows excellent results for the 3 unrelated external datasets, with sensitivity of 74%-98% and specificity of 69%-85%.

**Table 2:** Prediction on external validation sets.

Set	Set Size	In Bitter Domain	Pos	Neg	TP	FP	TN	FN	Sensitivity	Specificity
Bitter New	29	23	23		17			6	0.74	
UNIMI set	56	56	23	33	18	5	28	5	0.78	0.85
Phyto. Dictionary	93	75	49	26	48	8	18	1	0.98	0.69

Pos- number of positive molecules in the set. Neg- number of negative molecules in the set. TP- number of positive (bitter) molecules correctly classified. FP- number of negative (non-bitter) molecules incorrectly classified. TN- number of negative (non-bitter) molecules correctly classified. FN- number of positive molecules (bitter) incorrectly classified. Sensitivity TP/(TP + FN). Specificity TN/(TN + FP).

II) Validation by literature mining. We assumed that information about bitterness or other off-tastes of orally administered clinical drugs would be readily available in the literature or on the web.

BitterPredict was applied to DrugBank set of FDA approved drugs^46^ and the compounds were sorted by their predicted bitterness score. The names of the top 30 compounds (most likely to be bitter) and bottom 30 compounds (most likely to be non-bitter) were submitted to datamining in scientific publications (using Google Scholar), chemical databases (PubChem^47^ and ChemSpider^48^) and in the web (using Google) with the word “taste” or “bitter taste” to get an indication of their taste.

For the top predicted bitter compounds, 14 were described as bitter, 4 were indirectly described as bitter (for example the tablets that mainly include the compound of interest are described as bitter), 4 had description of unpleasant taste, for 8 no relevant data on taste could be found. Notably, none of the “predicted to be bitter” compounds had taste description other than bitter or unpleasant.

For the top predicted non-bitter compounds: for 6 molecules (20%) notion that they are bitter or might be bitter was found. For 6 molecules (20%) taste description other than bitter (“tasteless”, “mint”, “sweet with bitter aftertaste” and “a known bitter masking agent”) was found. For the majority of the predicted to be non-bitter molecules (18 molecules, ~60%) no indication of taste could be found. Since bad taste of a drug is a typical complaint, we assume that lack of mention of bitter taste indicates these are not very likely to taste bitter.

**Figure 4:**
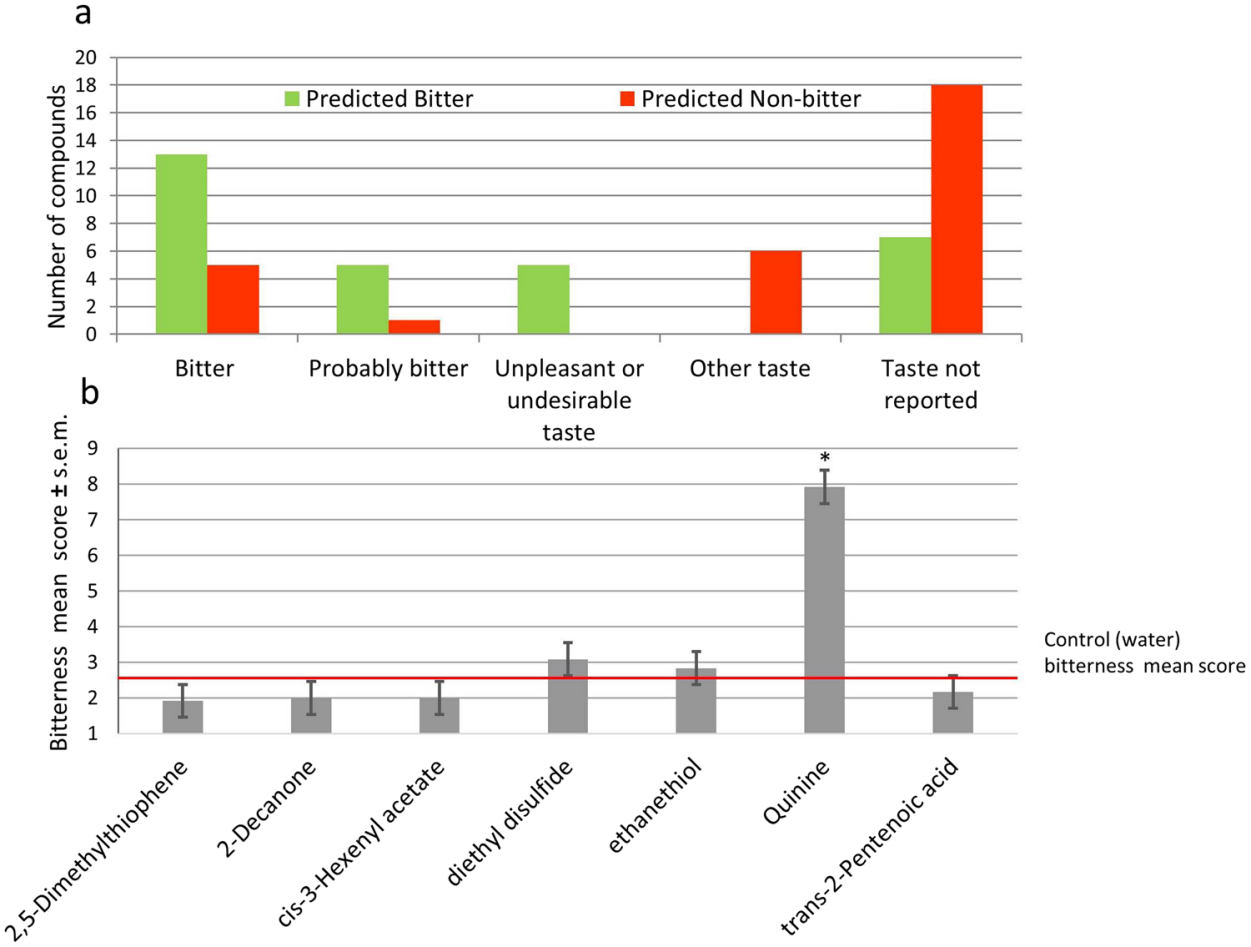
BitterPredict Validation: (**a**) DrugBank literature-derived information: histogram showing the taste description found in datamining for the 30 most likely bitter compounds and the 30 most likely non bitter compounds according to BitterPredict predictions. (**b**) Sensory evaluation of compounds predicted to be non-bitter: Bars indicate mean ± s.e.m. The red horizontal line represents the mean of water bitterness (control). Asterisks indicate a significance difference (P < 0.05) from control by the two-tailed Dunnett test.

Overall, these results indicate that ~60% of top predicted to be bitter drugs were mentioned to have bitter taste, while only 20% of predicted to be non-bitter had potential mention of bitter taste (Fig. 4a).

III) Validation by taste tests: To allow for sensory testing using human panel, BitterPredict was applied to Sigma-Aldrich Ingredients Catalog Flavors& Fragrances food which include Food-grade, Natural, Kosher or Halal materials. Among the 279 compounds extracted from Sigma-Aldrich flavors and fragrances catalog (http://go.sigmaaldrich.com/ff-catalog-download-safcglobal), only 14 were predicted bitter with score above 0.6. For 4 of these, molecules with the same name were already included in the BitterDB, and almost all the others were either insoluble, allowed for consumption only at very low concentration, or not readily available for purchase (see Supplementary file validation.xls for details), and were not tested. 105 compounds were predicted as non-bitter with score < −0.7. 6 compounds were chosen for sensory testing according to their prediction score, safety considerations and availability. The 6 non-bitter compounds were diluted in distilled water (0.5 mM) and tested in a sip&spit experiment by a panel of 12 subjects (see Methods for details). During the experiment the participants used nose clips to prevent smelling of solution odor. 5 out of the 6 tested compounds (83.3%) were rated equally or less bitter than the water used for dilution. Using the Dunnett test (alpha = 0.05) none of the 6 solutions differed significantly in bitterness from water (the significance for each of these comparisons was p > 0.8). Quinine, that was added to the experiment as an example of a known bitter compound, was significantly more bitter than water (the actual significance for this comparison was p < 0.0001) (Fig. 4b).

In summary, all the validation approaches suggest a reliable and consistent performance of BitterPredict for both bitterness and non-bitterness prediction.

### Model interpretation

**Figure 5:**
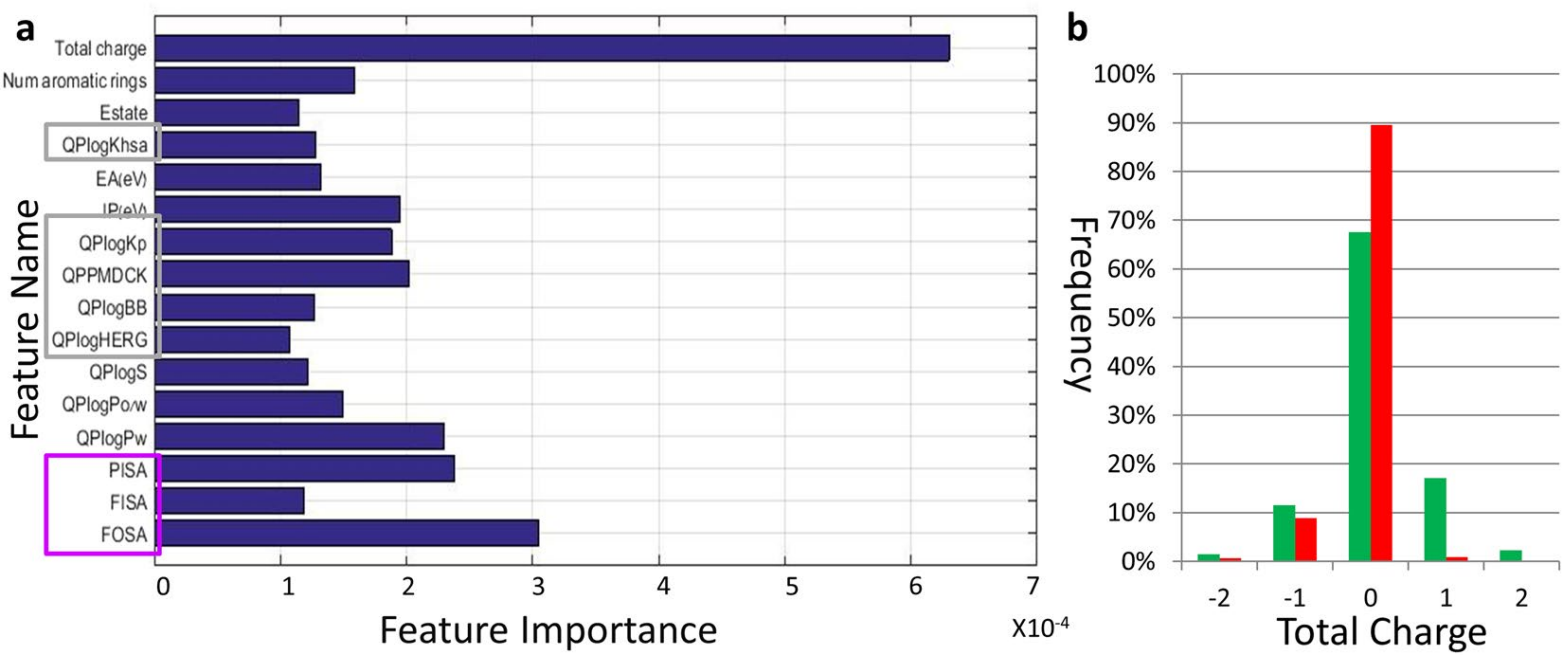
Descriptors contribution to The AdaBoost model (**a**) 16 descriptors with the most significant contribution to the AdaBoost model (contribution score greater than 1*10^−4^). (**b**) Total charge distribution in the bitter and non-bitter sets.

It is interesting to explore the contribution of the different descriptors to the model performance. This can be calculated (see Methods for details) from the contribution of the descriptor to reducing the error. Figure 5 shows the top 27% descriptors according to this analysis. The most important descriptor is the total charge. Indeed, the majority of the positively charged molecules are bitter molecules containing ammonium ion at physiological pH (Fig. 5b), suggesting that molecules with positively charged ammonium ion are more likely to be bitter than neutral or negatively charged ones.

Additional descriptors of importance are FOSA, FISA and PISA, which describe different parts of the molecular surface and are related to hydrophobicity.

QplogHERG, QPlogKp, QPPMDCK, QplogKhsa, QPlogBB (blockage of Human ether-a-go-go-related gene (hERG) K^+^ channels, skin permeability, transport in the gut blood barrier, binding to human serum albumin and blood-brain barrier transport measure) are connected to potential toxicity of the molecule in the body, and were found to have a relatively large contribution to the prediction model. Bitter molecules are enriched (22%) with compounds which are predicted to block hERG K^+^ channels compared to the negative set (10%) (see Supplementary Fig. S5). This result is in accord with our recent observation^24^ that >20% of the bitter tastants in BitterDB are known inhibitors of the hERG K+ channels. It was recently found that bitter receptors are also expressed in heart tissue^49^, thus some compounds could be acting on both bitter taste receptors and hERG channel in the heart tissue. Interestingly, QikProp descriptors related to potential toxicity tend to have larger contributions to the prediction model than descriptors related to specific molecular property (such as number of ring atoms, number of carboxylic acid group etc.).

### Prospective predictions

The BitterPredict classifier was next applied to several datasets of interest. Specifically, we used FooDB (http://foodb.ca/), a dataset of food constituents, DrugBank approved set^46^, natural product subset in ZINC15 database^50^ which holds commercially available secondary metabolites, and ChEBI^29^ as a representative set of random molecules. The predictions provide an estimate on the percentage of bitter molecules within related chemical datasets and are available via Supplementary files and via Ambinter website (http://www.ambinter.com/moleditor/web/), details in Data availability section below.

The estimation of the percentage of predicted bitter molecules in the different datasets are detailed in Table 3.

**Table 3:** Prospective predictions.

Data\Sets	FooDB	DrugBank approved	ChEBI	Natural Products
Number of molecules ready for prediction	20,661	1,553	37,033	28,217
Number of molecules in Bitter Domain	13,588	1,375	27,015	27,474
Predicted as bitter (% from molecules ready for prediction)	7926 **38.36**%	1,024 **65.94**%	16,188 **43.71**%	21,786 **77.21**%

The prediction on ChEBI represents the estimation of abundance of bitter compounds among random molecules. Interestingly, BitterPredict suggests that ~43% of random molecules may have some bitter taste. This means that automatic assumption that a random molecule is tasteless may be wrong in many cases.

Since ChEBI includes many synthetic molecules, it is of particular interest to evaluate the percentage of bitter compounds also among natural products. The natural products set used in this study contains secondary metabolites mainly from plants. Plants produce secondary metabolites including toxic chemicals as part of their defense system against herbivore attack, and many of these compounds are known to be bitter^51,52^. Indeed, 77% of the natural products library was predicted to have bitter taste. The high percentage of predicted bitter compounds in this set suggests that bitter taste may be among the most abundant tastes encountered in nature.

Many drugs are well known to have bitter taste^12,14,24^. In accord with this notion, 66% of the DrugBank library were predicted to be bitter.

Food ingredients represented in the FooDB, are predicted to include 38% bitter compounds. The relatively high percentage of bitter ingredients in FooDB is somewhat surprising, but may reflect the presence of many glucosinolates, terpenes, flavonoids and some alkaloids (such as quinine and caffeine) that are commonly consumed in foods and beverages such as coffee, tea, tonic water, vegetables and fruits, despite their bitterness.

To conclude, BitterPredict suggests that for an arbitrary small molecule there is 40% chance that it would elicit at least some bitter taste. For a molecule that belongs to a set that is more related to bitterness (such as drugs and natural products) the chances for some bitterness are much higher, around ~70%.

### Accessibility

Access to results and to BitterPredict software is detailed in the Data Availability section.

## Discussion

In this study, we have extended the dataset of bitter molecules and established a dataset of 2000 non-bitter molecules. Using these datasets, we have developed a bitter/non-bitter classifier. The classifier’s performance was evaluated on several external sets, showing high and robust sensitivity and specificity (around 0.8). Furthermore, datamining of top predictions in DrugBank set of clinical drugs and sensory experiment on food and flavor ingredients further confirmed the excellent performance of the predictor.

Application of BitterPredict to random molecules suggests that ~40% of the small molecules chemical space (ChEBI data set) have some bitter taste. Considering predictions with confidence score above 0.6 still predicts 11,115 (~30%) as bitter. This result is opposed to earlier assumption that random molecules are probably not bitter^16^.

To study questions related to taste perception and its shaping through evolution^53,54^, natural compounds (rather than molecules synthetized by chemists during the last centuries) should be studied. Therefore, it is particularly interesting to estimate the percentage of bitter compounds among natural products. Our results suggest that above 77% of natural products have some bitterness.

BitterPredict highlights the total charge, the surface related properties, and the potential toxicity of the molecule properties as important descriptors  differentiating bitter from non-bitter. In former studies, the connection between bitterness and hydrophobicity of peptides was discussed and disputed^23,55,56^. In accord with previous studies^20,57^, hydrophobicity was not found to be a predictive feature of bitterness, with similar AlogP distribution of bitter and non-bitter sets within the Bitter Domain (see Supplementary Fig. S1). However, low values of AlogP (<−3) are rare in the positive set, while 25% of the sweet molecules set have AlogP < −3. This means that very hydrophilic compounds are unlikely to be bitter. In addition, there are differences in the distributions of the important features indirectly related to hydrophobicity such as FOSA and hydrophilic component of total solvent accessible surface area (FISA). The combination of properties lends the classifier the ability to discriminate between the bitter and the non-bitter molecules.

The major advantages of BitterPredict is its high accuracy (~80%) and the ability to predict both bitterness and non-bitterness of a molecule based on its structure. The predictions are given a score, enabling the user to filter predictions according to intended use: for example such that false positive bitter predictions are rare, or such that false negative bitter predictions are rare. The relatively high sensitivity and specificity of BitterPredict enables exploration of large chemical spaces. The performance can be easily improved as more experimental data becomes available. The datasets established here are available to the users and will serve as a benchmark for further developments of bitterness prediction and classification methods.

There are also some limitations of the method that should be kept in mind: the method is applicable only to compounds within the Bitter Domain; scarcity of available data on levels of bitterness does not yet allow the discrimination between strongly vs. weakly bitter compounds.

Future studies will aim to classify the bitter space in further sub-categories in order to differentiate between strongly bitter vs. weakly bitter compounds, and between bitterness of compounds from different sources and habitats. The current analysis allows design of experiments on molecules outside of the current applicability domain, which will eventually lead to extension of the Bitter Domain.

To the best of our knowledge, this is the first study that attempts to estimate the proportion of bitter molecules in a general chemical space and in specific chemical datasets. The possible applications of the BitterPredict classifier include studies of basic questions related to evolution of taste in different species, bitter taste receptors de-orphanization, and practical applications in food and drug development.

## Methods

### Data Sets preparation

The largest fragments of the chemical structures from all sets were uploaded to Maestro (Schrödinger Release 2015–4: MS Jaguar, Schrödinger, LLC, New York, NY, 2015). Salt ions, peptides, inconsistencies, and structures with less than 3 atoms were removed. 3D structures and protonation states at biological pH 7.0 ± 0.5 were generated with Epik and LigPrep (Schrödinger Release 2015-4: LigPrep, Epik, LLC, New York, NY, 2015) retained the original chirality of the compound (if specified). For each molecule, the conformer with the lowest energy was extracted. In case a molecule has two protonation states in the defined pH, both molecules were included. Molecules that cannot be neutralized were removed from this study due to QikProp descriptors calculation limitation. Duplicates (i.e molecules with identical values for each one of the descriptors used) were also removed.

The sets were filtered to include only structures within the primary applicability domain, named, “Bitter Domain”: MW >700 and −3 <alogP <7 which was defined in this study (see Results section).

### Data sets

A summary of the different datasets used in this study are detailed in Table 4.

**Table 4:** Datasets in use.

	Name	Date of version update	Number of molecules in the original set	Number of molecules after database preparation procedures	Number of molecules in Bitter Domain	Ref
Positive set	BitterDB	05/2015	687	645	632	3
	Additional bitter	Nr	74	60	59	30
Negative set	Non-bitter in-house	Nr	1,573	1,531	1,451	
	Sweet	Nr	414	414	336	30
	Tasteless	Nr	135	133	130	30
Validation sets	UNIMI	Nr		56	56	
	Bitter New	Nr		29	23	
	Phyto. Dictionary	Nr		89	65	45
Perspective prediction	DrugBank approved	4/2014	1,621	1,553	1,375	46
	FooDB	1/2016	24,399	20,661	13,588	
	ChEBI	4/2015	44,651	37,033	27,015	29
	Natural Products from ZINC15	08/2016	38,469	28,217	27,474	50
	Sigma Ingredients Flavors & Fragrances	06/2016	1047	279	264	

Nr: not relevant.

Data sets used to build and analyze training and test sets:

Bitter Set (positive): Include mainly molecules from BitterDB^3^, a database of almost 700 bitter compounds which were described as bitter in the literature, or were reported experimentally as capable of activating at least one human bitter taste receptor: 632 molecules from this database used for building the classifier. In addition 59 unique molecules from Rojas *et al*.^30^ study were added to the positive set.

Non-Bitter Flavors: The dataset consists of 92 molecules which were cited in TOXNET^33^ as tasteless. 68 additional molecules were extracted from publications manually or using Marvin 6.1 naming capabilities, 2013, ChemAxon (http://www.chemaxon.com), 1,413 ‘probably not bitter’ molecules which were collected from Fenaroli’s handbook of Flavor ingredients^32^ in an automated fashion: compound was considered as non-bitter if the word bitter does not appear in its description fields. For these compounds the CAS numbers or generic names were manually extracted and used to look up the structures in PubChem^47^. Additional manual validation for the Fenaroli’s handbook molecules was performed by looking for the taste description of randomly chosen 30 molecules in other resources. We discovered only one molecule with unclear taste, and removed it. In addition, some amino acids were removed from this set due to conflicting description of their taste in different resources. Set size after data curation in Bitter Domain was 1,451.

Sweet and Tasteless sets: 414 sweet compounds, and 130 tasteless compounds that were reported as sweet or tasteless in taste experiments, where extracted from recent study by Rojas *et al*.^30^. After removing duplicates with other non-bitter subsets and dataset preparation procedures the set sizes were 336 for sweet and 130 tasteless.

### Evaluation sets

Hold out sets that were not used in training and testing routines.

Bitter New: 29 molecules that were collected recently from different publication^39–44^ to expand BitterDB and were not part of the train or test set. 23 molecules from this set that were in Bitter Domain were used for validation.

UNIMI set: Molecules that were synthesized during years 1990 to 2000 as part of studies on taste active compounds, usually sweeteners. The compounds were submitted to preliminary tasting trials with a panel of 5–8 untrained panelists. The informed consent was insured by the responsible principal investigator (A. Bassoli, University of Milano), and the taste sessions were carried out following the common procedures at the time^61^.

Phytochemical Dictionary:^45^ The dictionary includes 3,000 bioactive compounds from plants, for some of them a taste description is provided. The CAS numbers or generic names were manually extracted and used to look up the structures in PubChem^47^ or ChemSpider^48^. After data curation, 55 bitter and 39 non-bitter compounds were available, with 49 bitter and 26 non-bitter compounds in the Bitter Domain.

### Data set used for sensory evaluation

Sigma Ingredients Flavors & Fragrances: 1047 molecules where extracted from Sigma-Aldrich flavors and fragrances catalog (http://go.sigmaaldrich.com/ff-catalog-download-safcglobal) using Marvin 16.2.1 naming capabilities, 2016, ChemAxon (http://www.chemaxon.com). A large portion of the original data set (>50%) was identical to the Non-Bitter Flavors. After data curation, and removing compounds which are identical to compounds in the positive or negative set, 279 compounds were available, with 264 compounds in the Bitter Domain.

### Data set used for prospective prediction

DrugBank approved^46^: The data set includes 1,621 Food and Drug Administration (FDA) approved small molecule drugs. After data curation, 1,553 molecules were ready for prediction, with 1,375 molecules inside Bitter Domain.

FooDB: (http://foodb.ca/) A data set which holds tens of thousands of food constituent molecules. 24,399 molecules extracted from the FooDB SQL version. After data curation, 20,661 molecules where ready for prediction, with 13,588 molecules in Bitter Domain.

Natural Products Dataset: A data set which holds 38,469 commercially available natural products and excludes the ZINC15 *primary metabolites* subset (was downloaded from ZINC15^50^). The data includes several datasets, among them are herbal and plants natural products sets^58,59^. After data curation 28,217 molecules where ready for prediction, with 27,474 molecules in Bitter Domain.

#### PCA

PCA was used in order to estimate the variance and chemical space of the datasets that were used in this study. Prior to the PCA analysis, the descriptors were normalized such that each descriptor has zero mean and unit variance. The PCA was performed using Matlab (version R2015a; Mathworks, Inc., MA, USA), using the 2D structures of the molecules and 12 basic physiochemical descriptors calculated with Canvas (Schrödinger Release 2015-4: Canvas, Schrödinger, LLC, New York, NY, 2015): molecular weight (MW), lipophilicity (ALogP, the atomic LogP), rotatable bonds count (RB), polar surface area (PSA), electrotopological states (estate), molecular refractivity (MR), molecular polarizability (Polar), hydrogen bond acceptor (HBA), hydrogen bond donner (HBD), rings count (ring), chiral centers count (chiral) and heavy atoms count (HA).

### Descriptors

In addition to the 12 physicochemical descriptors detailed above, the total charge, and the number of aromatic rings descriptors were calculated with Canvas (Schrödinger Release 2015-4: Canvas, Schrödinger, LLC, New York, NY, 2015). 47 absorption, distribution, metabolism, and excretion (ADME/Tox) descriptors were calculated with QikProp module (Schrödinger Release 2015-4: QikProp, Schrödinger, LLC, New York, NY, 2015). The ‘predicted maximum transdermal transport rate descriptor’ (JM) from QikProp package was excluded due to high variance in values for very similar compounds, the HBA and HBD descriptors from the physicochemical descriptors were removed due to redundancy with QikProp descriptors. In total, 59 descriptors were used to build the model; the complete list of descriptors is available in Supplementary Table S3.

### Predictive models

Preliminary models: Preliminary models were computed in WEKA explorer software version 3.6.10^60^ with three different algorithms: Sequential minimal optimization (SMO), logistic regression and random forest. The models were built using a preliminary version of the training sets: BitterDB as positive set and non-bitter flavors set with 2,000 diverse selected molecules from ChEBI as negative set. Class imbalance was addressed by oversampling the positive set.

Ensemble methods models: Fitensemble and TreeBagger algorithms from Matlab Machine Learning (ML) toolbox (version R2015a; Mathworks, Inc., MA, USA) were used to train different preliminary models.

The AdaBoost classifier was calculated with decision trees as weak learners as implemented in the fitensemble algorithm in Matlab. In order to avoid overfitting and improve performance several parameters were adjusted by examining their influence on the mean squared error (MSE) in the train set with respect to the MSE of the test set: i) Learning rate: was set to 0.15 (default is 1.0) ii) Number of trees: was set to 200 iii) Maximum number of splits in each tree (‘maxNumSplits’), and minimum number of samples in tree leaf (‘MinLeafSize’), were set to 15 (default is 5) and 5 (default is 1) respectively. The last two parameters restricted each decision tree depth.

The AdaBoost algorithm is calculated in several iterations, in each iteration a new decision tree is calculated and a weight vector is adjusted to learn from previous misclassified samples. Weights of the misclassified samples are increased while weights of the correctly classified samples are reduced. In each iteration the algorithm minimizes the weighted classification error (see equation 1, equation was adapted from^61^).

Equation 1: Weighted classification error^61^

1 $$\varepsilon t=\sum _{n=1}^{N}{d}_{n}^{(t)}I({y}_{n}\ne {h}_{t}({x}_{n}))$$

*x*_n_ is the descriptors vector for sample *n*,

*y*_n_ is the true label for sample *n*,

*h*_*t*_ is the prediction of the learner with index *t*,

I is the indicator function.

${d}_{n}^{(t)}$ is the weight of sample *n* at step *t*

### Prediction with AdaBoost

The predictions are made by calculating the weighted average of the predictions given by each tree in the ensemble. The weights for each tree are determined according to each tree weighted classification error, such that predictions from trees with lower weighted classification error get higher weight (see equation 2, the equation is adapted from^61^).

2 $$f(x)=\sum _{t=1}{\alpha }_{t}{h}_{t}(x)$$

where:

$${\alpha }_{t}=\frac{1}{2}log\frac{1-{\varepsilon }_{t}}{{\varepsilon }_{t}}$$

*X new data*

*h*_*t*_ is the prediction of the learner with index *t*,

### Imbalanced data and within-class imbalance

Two main issues need to be considered while designing the prediction model: Imbalanced and Within-class imbalance^62^.

Imbalanced data - The negative set is almost three times larger than the positive set. Machine learning approaches that aim to minimize the number of mistakes would result in overall high accuracy (accuracy = percentage of the correctly classified samples out of the total samples), even though the sensitivity (sensitivity = percentage of the correctly classified positive samples out of the total positive samples) will be low^62^. In our case, this would lead to predicting most of the samples as “non-bitter”. Our goal is to avoid this situation.

Within-class imbalance^62^ - The negative set is comprised of three subsets; non-bitter flavors, tasteless and sweet. The number of compounds in the tasteless and sweet subsets is much smaller than in the non-bitter flavors set, but they occupy a different chemical space.

Both issues are addressed in the final model by setting the weights in the initial observation weight vector. The observation weight vector length is the number of compounds; each compound gets a proportional weight. Weight vector was calculated according to compounds set size such that the sum of all bitter samples weight is 0.5 and the sum of all non-bitter samples weight is 0.5. The sum of weights in each non-bitter subset was set to: sweet: 0.16 tasteless 0.16 and flavors 0.18.

### Model performance

Datasets were randomly divided to train and hold out test set containing 70% and 30% of the molecules respectively, ensuring the original proportions of the different subsets (bitter, flavors, sweet, and tasteless). The different models were trained only on the train set, and the four parameters described above (learning rate, number of trees, ‘maxNumSplits’, MinLeafSize’) were adjusted by their performance in the training set and with respect to their performance in the hold out test set to avoid overfitting. The external validation sets were used only to assess the model.

Classification models were evaluated by means of sensitivity (*Se*) and specificity (*Sp*) of classes. *Se* describes the true positive rate i.e how many positive samples were correctly identified as positive. Sp describes the true negative rate i.e how many negative samples were identified as negative.

3 $$Sp=\frac{TN}{TN+FP\,}\,Se=\frac{TP}{TP+FN}$$

where TP, TN, FP and FN represent the number of true positives, true negatives, false positives, and false negatives, respectively.

Descriptor’s contribution to the model’s performance was calculated using the Predictor Importance procedure in the fitensemble function is Matlab (version R2015a; Mathworks, Inc., MA, USA)^63^. This procedure is summing the changes in the MSE in each tree between the original split MSE in the parent node and the total MSE for the two children.

### Sensory experiment for validation of predictions

The experiment included 12 participants (8 females, 4 males, mean age = 30.41, age range: 24–42), with no reported pregnancy, food intolerance, allergies or use of medication. The participants were instructed not to consume anything other than water for 1 h prior to the experiment. During the experiment the participants used nose clips. Using Compusense Cloud on-line software (Compusense Inc., Guelph, ON, Canada) the participants were guided to swish each one of the eight solutions (6 solutions of predicted non-bitter compounds, water and a known bitter compound quinine) for 5 sec. without swallowing. Between sample tastings, participants were instructed to rinse their mouth with water and wait 30 seconds. The order of the solutions was randomly assigned to each participant by the Compusense software. After sipping and spitting of each solution, participants had to evaluate its bitterness and sweetness by using 9-Likert Scale on Compusense Cloud. 9-Likert scale ranged from 1 (no sensation) to 9 (extremely strong sensation). In addition, participants had to report the dominant taste of each solution and any additional tastes they recognized. All research procedures were performed according to relevant guidelines and regulations and were ethically approved by the “Committee for the Use of Human Subjects in Research in The Robert H. Smith Faculty of Agriculture Food and environment, the Hebrew University of Jerusalem”, informed consent was obtained from all participants.

### Reagents

Ethanethiol, 2-Decanone, Diethyl disulfide, 2,5-Dimethylthiophene, trans-2-Pentenoic acid, cis-3-Hexenyl acetate and quinine sulfate were purchased from Sigma (CAS Numbers: 75-08-1, 693-54-9, 110-81-6, 638-02-8, 13991-37-2, 3681-71-8 and 207671-44-1 respectively). All compounds were dissolved to a final concentration of 0.5 mM in double distilled water (Millipore-filtered). The solutions concentration was selected to be 0.5 mM, higher than reported detection thresholds of known bitter compounds, (such as PROP and quinine^64^), and safe.

### Statistical analyses

Statistical tests were conducted using JMP Pro 13 (SAS). Data were first analyzed using ANOVA with participants as a random effect. Thereafter the Dunnett test was used to compare mean bitterness for each solution to the water stimuli. Significance was set at p < 0.05, and two-tailed tests were used where relevant.

### Data Availability

Training and test sets generated and/or analysed in the current study are available from the corresponding author upon reasonable request.

Matlab code for BitterPredict is provided via BitterDB http://bitterdb.agri.huji.ac.il/dbbitter.php#BitterPredict and via GitHub repository https://github.com/Niv-Lab/BitterPredict1. The allowed input is CSV or Excel file holding QikProp and physicochemical descriptors calculated with Schrödinger as listed in detail via Supplementary Table S3.

The external sets used for validation are included in the Supplementary file validation.xls online.

The prospective predictions on DrugBank, FooDB, ChEBI and ZINC natural products are included in Supplementary file prospective_prediction_sets.xls online and via Ambinter (http://www.ambinter.com) software website in the following links:

FooDB: http://www.ambinter.com/moleditor/web/display/c6ff1327

DrugBank: http://www.ambinter.com/moleditor/web/display/5fb411e8

ChEBI: http://www.ambinter.com/moleditor/web/display/6dbe122e

Natural Products: http://www.ambinter.com/moleditor/web/display/6bcc1278

Predicted molecules can be browsed and filtered by several descriptors. Users can download the data in different formats and search for similar compounds.

Summary of the supplementary data files can be found in Supplementary Table S1.

Requests for predictions for additional datasets can be submitted to the authors via email request.

## Electronic supplementary material

Supplementary data

Supplementary Dataset 1

Supplementary Dataset 2
