# Supplementary material for: Bitter or not? BitterPredict, a tool for predicting taste from chemical structure
Source: Sci Rep. 2017 Sep 21;7:12074. doi: 10.1038/s41598-017-12359-7 (PMC5608695; doi:10.1038/s41598-017-12359-7)
Supplement: Supplementary file 1 — Supplementary data [file 41598_2017_12359_MOESM1_ESM.pdf]

Supplementary data:

Bitter or not? BitterPredict, a tool for predicting taste from chemical structure

Ayana Dagan-Wiener[1,2], Ido Nissim[1,2], Natalie Ben Abu[1,2], Gigliola Borgonovo[3], Angela Bassoli[3], Masha Y. Niv[1,2]*

[1]Institute of Biochemistry, Food Science and Nutrition, The Robert H. Smith Faculty of Agriculture, Food, and Environment, The Hebrew University of Jerusalem, Rehovot, 76100, Israel.

[2]The Fritz Haber Center for Molecular Dynamics, The Hebrew University of Jerusalem, 91904, Israel.

[3] DeFENS-Department for Food, Environmental and Nutritional Sciences, University of Milan, Via Celoria 2, Milano, 20133, Italy.

*correspondence to masha.niv@mail.huji.ac.il

Table S1 Additional Supplementary files:

File Name	Description
validation.xls	1) Holds structures (smiles format), Bitter label (0-non-bitter , 1 bitter) and BitterPredict results for the three external sets used for validation (Bitter New, UNIMI set, Phyto. Dictionary).<br>2) Holds the Literature mining validation on the top 30 predicted bitter and non-bitter from DrugBank.<br>3)Holds the predicted bitter compounds (score>0.6) and predicted non-bitter compounds (score<-0.7) from Sigma-Aldrich Ingredients Catalog Flavors& Fragrances food with explanation on top predicted compounds that were not used for validation .
prospective_prediction_sets.xls	Holds structures (smiles format) and BitterPredict results for the four datasets used for the prospective predictions (FooDB, DrugBank,ChEBI, Natural Products ZINC15)

Table S2: Prediction results using only the non-bitter flavors as negative group.

Results of AdaBoost model which was trained only with non-bitter flavors as negative set.

Table 1.A shows good performance measure on the hold-out test set.

Table 1.B shows the high percentage predicted to be bitter in FooDB DrugBank and ChEBI.

A.	Sensitivity TP/TP+FN	Specificity TN/TN+FP	Accuracy (TP+TN)/Total
AdaBoost	0.75	0.88	0.84
**B.**	**FooDB**	**DrugBank**	**ChEBI (random)**
% molecules from set predicted to be Bitter	43.2%	80.8%	70.3%

TP- number of positive (bitter) molecules correctly classified.
FP- number of negative (non-bitter) molecules incorrectly classified.
TN- number of negative (non-bitter) molecules correctly classified.
FN- number of positive molecules (bitter) incorrectly classified.

<u>Figure S1:</u>

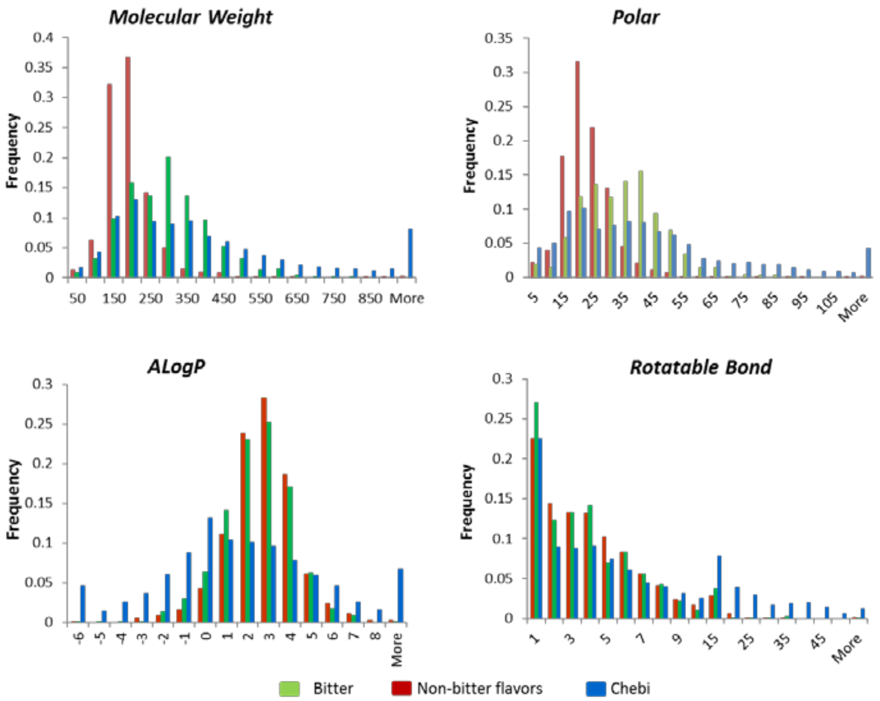

Figure S1: Comparison of selected physicochemical properties between the Bitter, Non-bitter flavors and ChEBI sets. In terms of The Molecular weight and Polarity the Non-bitter flavors set has low variance and occupies narrower range compared to the Bitter and ChEBI set. The hydrophobicity of the Bitter and Non-bitter flavors set, represented by AlogP, is similar and somewhat higher compared to random molecules (ChEBI). The distribution of number of rotatable bonds is similar between the three sets.

Figure S2:

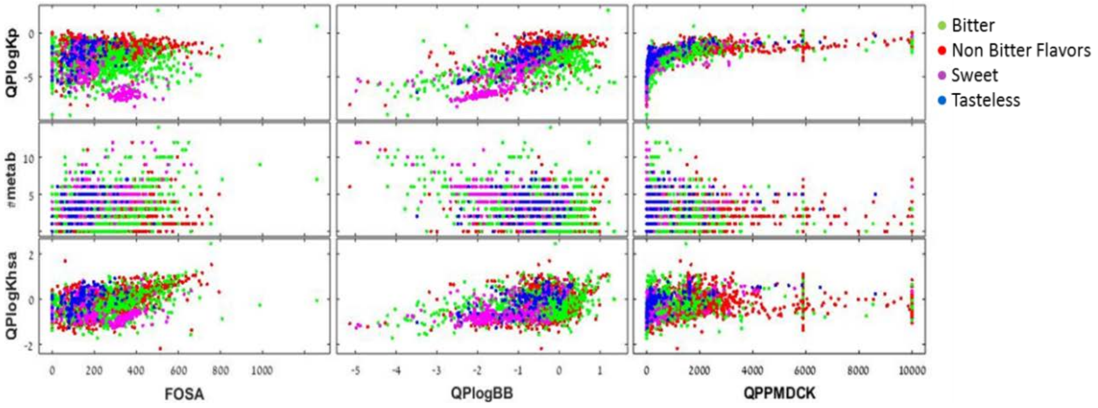

Figure S2: Bivariate statistical analysis of selected QikProp properties. The bivariate statistical analysis indicates combined properties ranges which are enriched with bitter molecules. Most of the bitter molecules properties tend to reside in the range that is suggested by QikProp (version 4.6 Schrödinger, LLC, New York, NY, 2015) as acceptable for drugs -3<QPlogBB<1.2, -1.5 < Prediction of binding to human serum albumin (QPlogKhsa)<1.5, -8< Predicted skin permeability (QPlogKp)<-1, Predicted apparent Madin-Darby Canine Kidney Epithelial Cells (MDCK) cell permeability in nm/sec. (QPPMDCK)>25, Number of likely metabolic reactions 0<(#metab)<8, Hydrophobic component of the total solvent accessible surface area (FOSA)<750 (for more details about the suggested ranges please refer to QikProp manual: http://gohom.win/ManualHom/Schrodinger/Schrodinger_2015-2_docs/qikprop/qikprop_user_manual.pdf)

Figure S3:

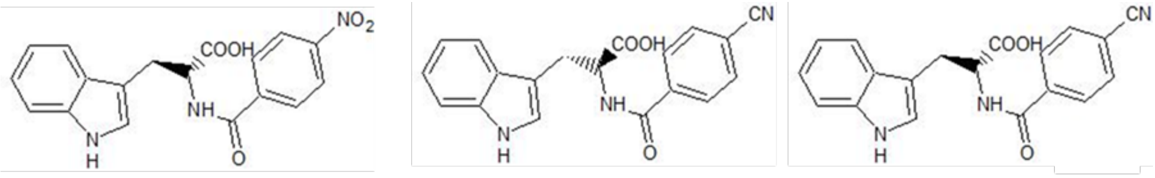

A) Bitter B) Tasteless C) Bitter

Figure S3: UNIMI set molecules: Examples of 3 molecules from the challenging UNIMI set, demonstrating that similar, molecules (A,B) and even stereo isomers (B,C) elicit different tastes. BittePredict predicts all three molecules as bitter, though B and C got relatively low bitterness score (<0.2)

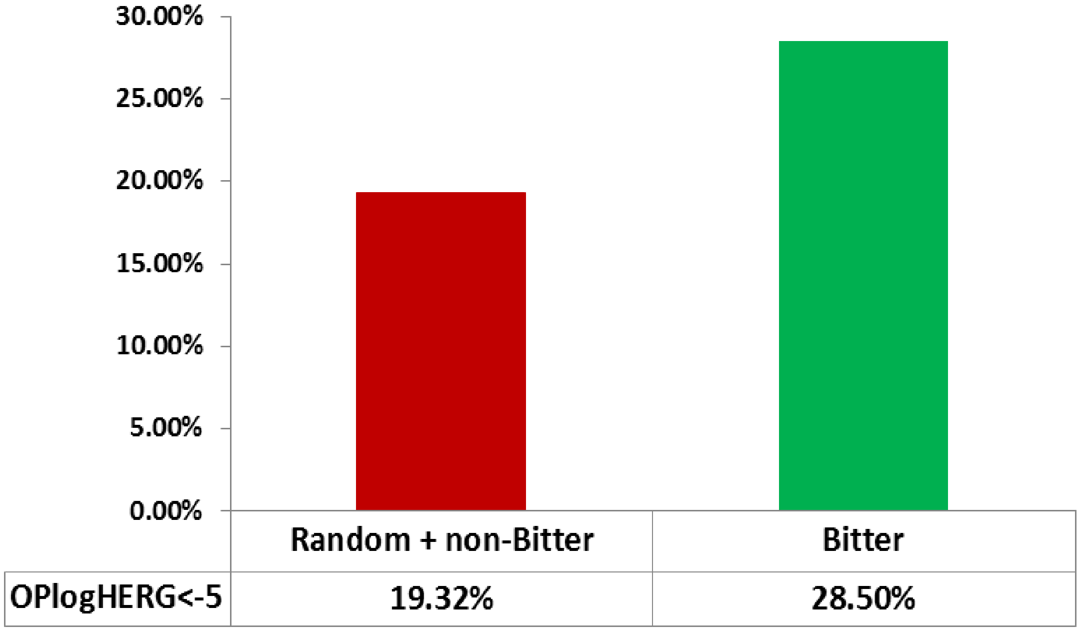

Figure S4: Predicted IC50 value for blockage of Human ether-a-go-go-related gene (hERG) potassium channels in the bitter and random+non bitter sets. We found that bitter compounds are enriched (~30%) with compounds which are predicted to block gene (hERG) K+ channels compared to random + non-bitter set (20%). The result fits with our recent finding that >20% of the bitter tastants in BitterDB are reported to inhibit hERG. It was recently found that bitter receptors are also expressed in heart tissue, which might be connected to this common off- target of bitter molecules.

<u>Table S3 descriptors used in BitterPredict classifier:</u>

47 descriptors from QikProp	**#stars-** Number of property or descriptor values out of 24 properties and QikProp descriptors hat fall outside the 95% % range of similar values for known drugs.<br>**#amine** Number of non-conjugated amine group<br>**#amidine** Number of amidine and guanidine groups.<br>**#acid** Number of carboxylic acid groups.<br>**#amide** Number of non-conjugated amide groups.<br>**#rotor** Number of non-trivial (not CX3), non-hindered (not alkene, amide, small ring) rotatable bonds.<br>**#rtvFG** Number of reactive functional groups;<br>**CNS** - Predicted central nervous system activity<br>**dipole†** Computed dipole moment of the molecule.<br>**SASA** Total solvent accessible surface area (SASA) in square angstroms using a probe with a 1.4 Å radius.<br>**FOSA** Hydrophobic component of the SASA (saturated carbon and attached hydrogen).<br>**FISA** Hydrophilic component of the SASA (SASA on N, O, H on heteroatoms, carbonyl C). 7.0 – 330.0<br>**PISA** π (carbon and attached hydrogen) component of the SASA.<br>**WPSA** Weakly polar component of the SASA (halogens, P, and S).<br>**volume** Total solvent-accessible volume in cubic angstroms using a probe with a 1.4 Å radius.<br>**donorHB** Estimated number of hydrogen bonds that would be donated by the solute to water molecules in an aqueous solution.<br>**accptHB** Estimated number of hydrogen bonds that would be accepted by the solute from water molecules in an aqueous solution<br>**dip^2/V†** Square of the dipole moment divided by the molecular volume.<br>**ACxDN^.5/SA** Index of cohesive interaction in solids.<br>**glob** Globularity descriptor.<br>**QPpolrz** Predicted polarizability in cubic angstroms.<br>**QPlogPC16** Predicted hexadecane/gas partition coefficient.<br>**QPlogPoct** Predicted octanol/gas partition coefficient.<br>**QPlogPw** Predicted water/gas partition coefficient.<br>**QPlogPo**/w Predicted octanol/water partition coefficient.<br>**QPlogS** Predicted aqueous solubility<br>**CIQPlogS** Conformation-independent predicted aqueous solubility,<br>**QPlogHERG** Predicted IC50 value for blockage of HERG K+ channels.

	**QPPCaco** Predicted apparent Caco-2 cell (Caco- 2 cells are a model for the gut-blood barrier, (non-active transport).<br>**QPlogBB** Predicted brain/blood partition coefficient.<br>**QPPMDCK** Predicted apparent MDCK (Madin-Darby Canine Kidney Epithelial) cell permeability in nm/sec.<br>**QPlogKp** Predicted skin permeability.<br>**IP**(ev) calculated ionization<br>**EA(eV)** calculated electron affinity<br>**#metab** Number of likely metabolic reactions.<br>**QPlogKhsa** Prediction of binding to human serum albumin.<br>**HumanOralAbsorption** Predicted qualitative human oral absorption<br>**PercentHumanOralAbsorption** Predicted human oral absorption on 0 to 100% scale.<br>**SAFluorine** Solvent-accessible surface area of fluorine atoms<br>**SAamideO** Solvent-accessible surface area of amide oxygen atoms.<br>**PSA** Van der Waals surface area of polar nitrogen and oxygen atoms and carbonyl carbon atoms.<br>**#NandO** Number of nitrogen and oxygen atoms.<br>**RuleOfFive** Number of violations of Lipinski's rule of five. The rules are: mol_MW < 500, QPlogPo/w < 5, donorHB ≤ 5, accptHB ≤ 10.<br>**RuleOfThree** Number of violations of Jorgensen's rule of three. The three rules are: QPlogS > -5.7, QP PCaco > 22 nm/s, # Primary Metabolites < 7.<br>**#ringatoms** Number of atoms in a ring<br>**#in34** Number of atoms in 3- or 4-membered rings<br>**#in56** Number of atoms in 5- or 6-membered rings<br>**#noncon** number of ring atoms not able to form conjugated aromatic systems (e.g. sp3 C).<br>**#nonHatm** Number of heavy atoms (non hydrogen atoms)
12 *Physiochemical* and topological descriptors calculated by Canvas and QikProp)	**MW** molecular weight,<br>**ALogP** lipophilicity,<br>**RB** rotatable bonds count,<br>**PSA** polar surface area,<br>**Estate** electrotopological states,<br>**MR** molecular refractivity,<br>**Polar** molecular polarizability ,<br>**Ar ring** aromatic rings count ,<br>**Ring** rings count,<br>**Chiral** chiral centers count ,<br>**HA** heavy atoms count ,<br>**Total charge**.
